# Pulmonary osseous metaplasia in association with renal cell carcinoma: A rare case report and literature review

**DOI:** 10.1016/j.radcr.2025.01.081

**Published:** 2025-03-08

**Authors:** Berun A. Abdalla, Fahmi H. Kakamad, Ari M. Abdullah, Sami S. Omar, Razan Babarasul Jalal, Harem K. Ahmed, Rezheen J. Rashid, Soran H. Tahir, Pavel Mustafa Kareem, Shvan H. Mohammed, Hemin S. Mohammed

**Affiliations:** a Scientific Affairs Department, Smart Health Tower, Madam Mittrand, Sulaymaniyah, Iraq; b Kscien Organization for Scientific Research (Middle East office), Sulaymaniyah, Iraq; c College of Medicine, University of Sulaimani, Sulaymaniyah, Iraq; d Department of Pathology, Sulaimani Teaching Hospital, Sulaymaniyah, Iraq; e Rizgary Oncology Center, Erbil, Iraq; f Department of Radiology, Ministry of Peshmerga, Shorsh General Hospital, Sulaymaniyah, Iraq; g Department of Medicine, Shar Hospital, Sulaymaniyah, Iraq; h Department of Oncology, Hiwa Hospital, Sulaymaniyah, Iraq; i College of Nursing, University of Sulaimani, Adult Nursing Department, Sulaymaniyah, Iraq

**Keywords:** Lung ossification, Uniport VATS resection, Osseous metaplasia, Renal cell carcinoma

## Abstract

Pulmonary osseous metaplasia (POM) is a rare occurrence often discovered postmortem, involves the development of fully formed bone tissue within the lung parenchyma. The aim of this study is to present a case of pulmonary osseous metaplasia in a 66-year-old female. A 66-year-old woman previously diagnosed with renal cell carcinoma (RCC) was referred for a pulmonary nodule discovered during a CT scan due to chest pain and shortness of breath. Clinical exams were normal, and the CT revealed solid nodules suspected to be metastatic. The patient underwent uniport video-assisted thoracoscopic surgery (VATS) for nodule resection, revealing metastatic disease and myeloid-osseous metaplasia in histopathological examination. After a 4-day hospital stay with intravenous antibiotics, the postoperative period was uneventful. The exact etiology of POM remains unclear, but it is thought to be associated with chronic inflammation and irritation of the lung tissue. The diagnosis of POM typically requires histopathological confirmation, and treatment options are limited due to its rarity. Myeloid osseous metaplasia of the lung is an exceedingly rare occurrence, and it may be associated with renal cell carcinoma.

## Graphical Abstract

**Figure:**
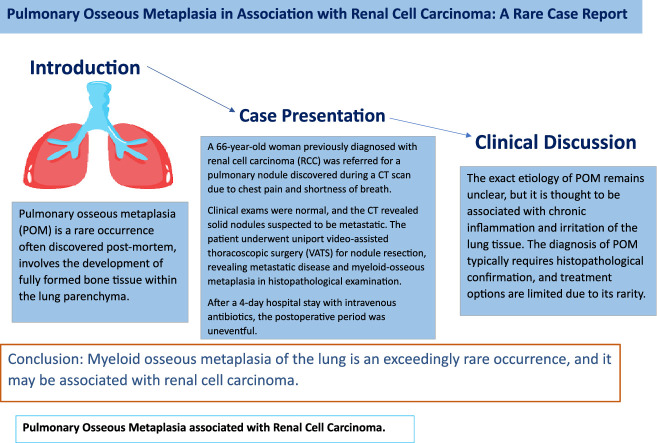

## Background

Pulmonary osseous metaplasia (POM), also known as ossification of lung, refers to the formation of fully developed tissue of bone within the lung's parenchyma. This condition is infrequent and typically occurs in conjunction with other chronic lung ailments, such as bronchiectasis, pneumonia, and lung fibrosis [1,2]. The incidence rate of POM is approximated to be between 0.2 and 0.4 occurrences per 100,000 individuals per year. The incidence rate is higher in older adults and in men [3]. Typically, POM is detected postmortem during autopsies and is rarely diagnosed during a patient's lifetime [4]. The extent of bone distribution in the lungs can vary, with some cases limited to specific areas [1]. Unless accompanied by other medical conditions, individuals with this condition usually exhibit no symptoms, and its diagnosis often necessitates the use of various imaging techniques and bone scans [2].

Disseminated lung ossification generally presents in 2 primary patterns: the dendriform pattern, marked by branching along the terminal airway with marrow islands, and the nodular pattern, which is more frequently observed in the space of alveoli and tends to be more confined [4]. While the exact pathophysiology remains poorly understood, some studies in the literature suggests an association between this condition and other lung diseases [2]. Given the rarity of this condition, there is limited information available regarding its gender prevalence or preferred age group. It is noteworthy that most reported cases of general osseous metaplasia have been in females [5].

The current study aims to report a case of POM in a female individual aged 66-year-old.

## Case presentation

### Patient information

A female aged 66-year-old was diagnosed previously with renal cell carcinoma of the left kidney. She was referred for management of the pulmonary nodule which found in the computed tomography (CT) scan after she had chest pain and shortness of breath.

### Clinical findings

The examination of respiratory, cardiovascular, and genitourinary tract was normal. The vital signs were normal. There were no indications of tuberculosis (TB) and her past medical history was negative regarding TB.

### Diagnostic assessment

**Fig. 1:**
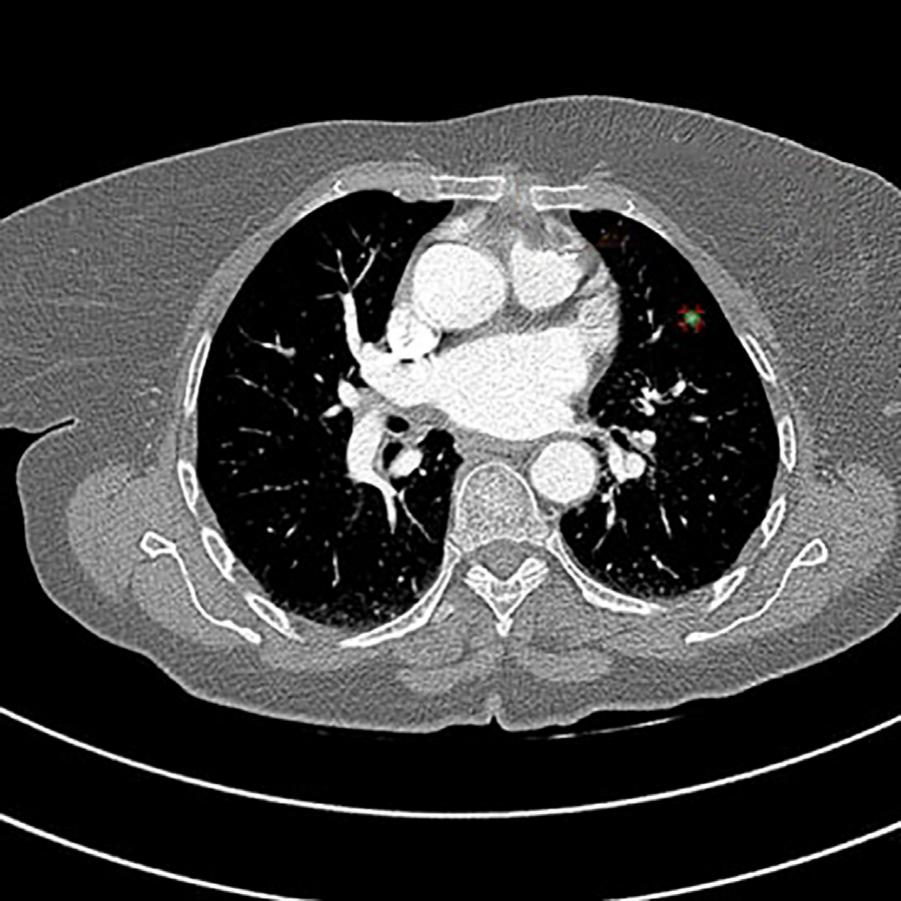
Axial HRCT chest showing well-defined lobulated left lower lobe pulmonary nodule, measuring 137 HU in density, with no calcification, suspicious of metastasis.

**Fig. 2:**
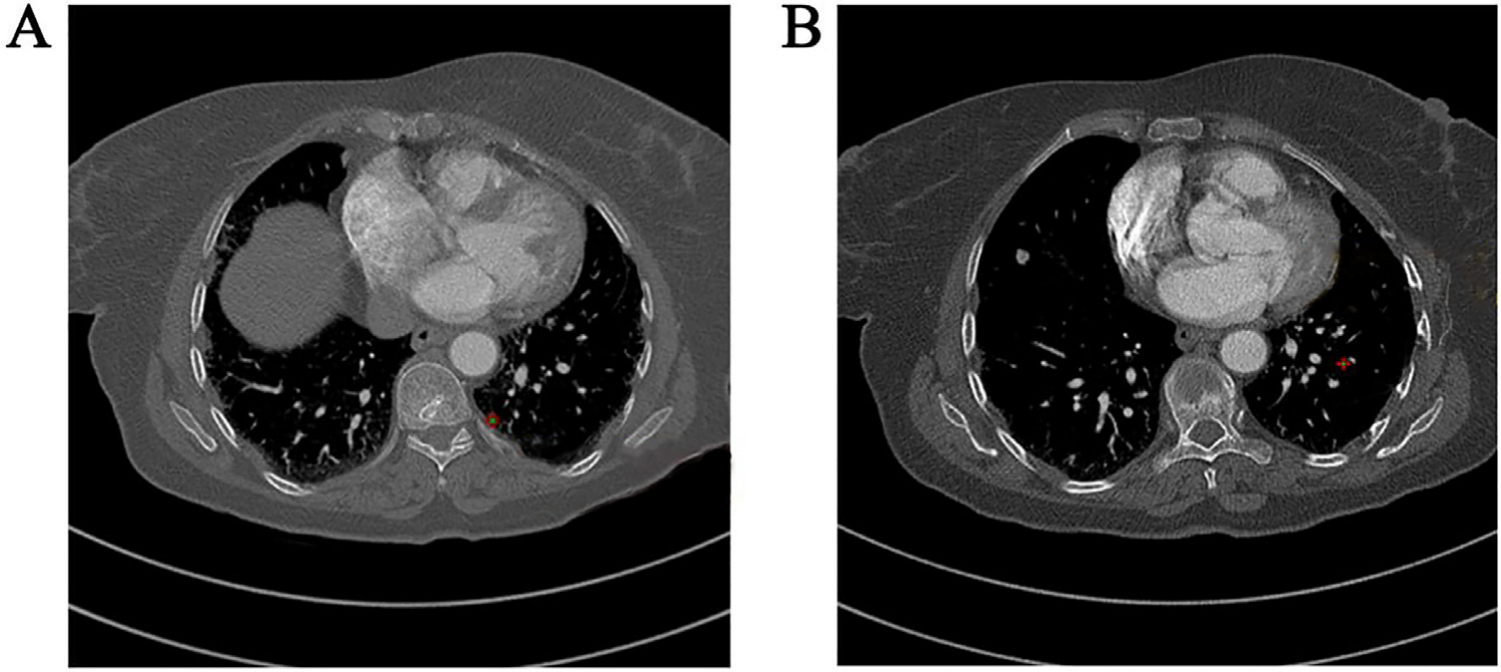
Chest CT scan showing (A) small nodule in the left lower lobe with faint calcification, measuring 476 HU in density, (B) Small nodule in the left lower lobe with frank calcification, measuring 880 HU in density.

A chest CT scan showed a clearly defined solid hyperdense nodules in the anterior part of the left lower lobe. The largest nodule measured 9 × 7 mm, with a density of 137 HU and showed no evidence of calcification. The radiologist presumed metastasis (Fig. 1). Two additional smaller nodules were identified in the left lower lobe. The first nodule demonstrated faint calcification with a density of 476 HU, while the second nodule exhibited frank calcification with a density of 880 HU (Fig. 2).

### Therapeutic intervention

**Fig. 3:**
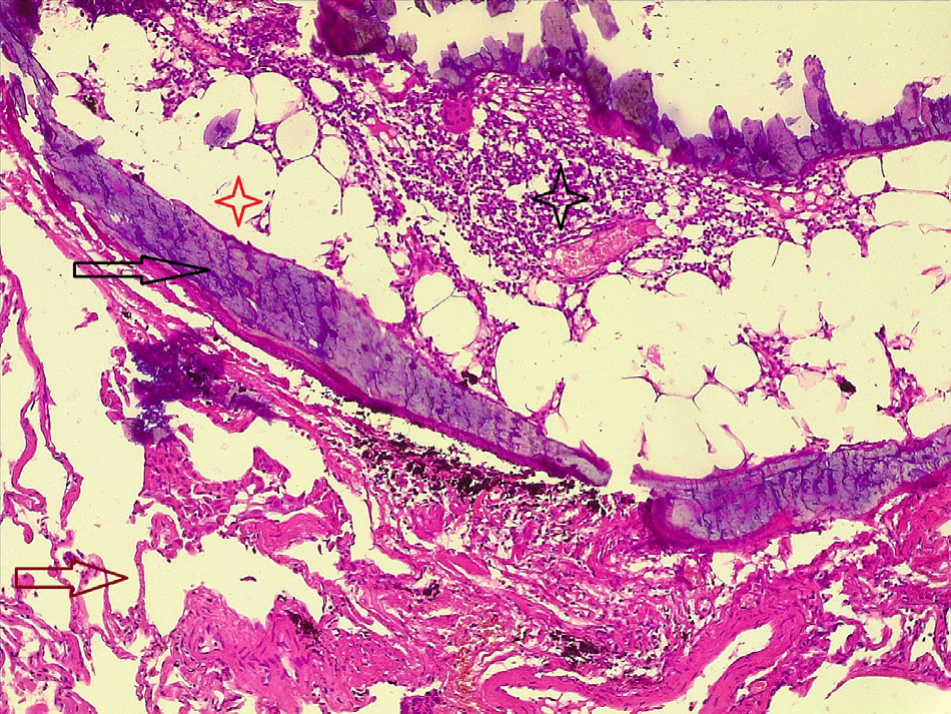
Section shows lung parenchymal tissue composed of alveoli (brown arrow), contains a nodule that composed of bone trabeculae (dark arrow), within the bone there is marrow elements (dark star) and adipose tissue (red star).

After diagnosis, she underwent preparation for general anesthesia, the nodules marked by a dye through CT guided before operation. Positioned laterally, the nodules were excised through a uniport video-assisted thoracoscopic surgery (VATS). Histopathological examination of one specimen indicated metastatic diseased cells, while 2 other nodules showed myeloid POM (Fig. 3).

### Follow-up and outcomes

The female patient stayed for 4 days in the hospital. During this period, she received intravenous antibiotics and analgesics. The postoperative period was free from complications.

## Discussion

The identification of fully formed bone tissue within the parenchyma of the lung is an unusual occurrence [5]. Pulmonary calcifications are frequently observed in various systemic disorders, with calcium deposition as a reaction to prolonged inflammation [2]. The transformation of pulmonary fibroblasts into osteoblasts may lead to pulmonary ossification, and the precise mechanisms behind these processes are not fully comprehended. In instances of widespread pulmonary ossification, the reaction is likely a protective response to persistent irritation and damage [4]. Osseous metaplasia, a condition stemming from chronic inflammation, is influenced by risk factors such as cigarette smoking, infections, environmental pollutants, chronic aspiration, and certain drugs. Nevertheless, the progressive nature of the disease is probably influenced by a genetic predisposition, as none of these factors alone adequately accounts for the disease's advancement [6]. Notably, despite its linking with infections of the lung, lungs diffuse dendriform ossification was identified in 5 out of 75 patients who previously diagnosed with interstitial pneumonia confirmed through biopsy [7]. Osseous metaplasia's nodular pattern, as evidence in this case, is even rarer.

Due to the scarce information in the genuine literature concerning POM, its precise causes and underlying mechanisms remain uncertain. Ossification has been noted in bronchial cartilage which is a vascular, alongside other abnormalities like ingrowth calcification and fibrovascular, particularly in individuals who have underwent transplantation of lung [8]. Bacterial infections resulting from cystic fibrosis, which can lead to the elimination of bronchial cartilage, have also been suggested as a potential inducer of osseous metaplasia [9]. Lung bone metaplasia has been documented in individuals with infections of TB [10]. Prior lung injury, hypercalcemic conditions, and environmental conditions which elevated levels of pH are among the other factors implicated in the etiology of the condition [2]. The basic environment resulting from injury to scar tissue facilitates the calcium deposition [11].

A definitive diagnosis necessitates confirmation histopathologically, although a CT scan can provide suggestive evidence of the condition [1]. The dendriform pattern manifests as branching shadows characterized by dense calcification, often resembling fibrosis and bronchiectasis stemming from a scar, while the subpleural nodular pattern, characterized by multiple nodules less than 1 cm, is indicative of an infection previously [3]. In the present case, the individual exhibited the nodular subtype, featuring several enhancing basal lung nodules, all measuring less than 8 mm. The process of diagnosis was ultimately confirmed through histopathological examination, revealing small calcified nodules in the parenchymal tissue containing mature bone trabeculae with marrow elements within their spaces of medulla.

Due to the rarity of the condition, there is no universally agreed-upon approach documented in the literature for its management [1]. In this specific case, after a 6-week observation period, a repeat CT scan, and a consultation with the tumor board, a provisional diagnosis of pulmonary metastasis from Renal cell carcinoma was made, leading to the recommendation of uniport VATS resection [12]. It is important to note that all references cited in this report have been thoroughly scrutinized for credibility [13]. Notably, this report has limitations, including the absence of data that hinders the exploration of possible development of pulmonary calcification as a treatment side effect. Furthermore, the case lacks details on levels of calcium in blood, blood gas, vitamin D, and pH.

## Conclusion

Myeloid POM is an exceedingly infrequent occurrence, and it may be associated with renal cell carcinoma.

## Consent for publication

Not applicable.

## Availability of data and material

All data and materials are kept by the first and corresponding authors.

## Patient consent

Consent has been taken from the patients and the family of the patients
